# Supplementary material for: Exploring the association between stress-related hormonal changes, behaviours and facial movements after an interval training exercise in French Standardbred
Source: PLoS One. 2025 Nov 5;20(11):e0328430. doi: 10.1371/journal.pone.0328430 (PMC12588519; doi:10.1371/journal.pone.0328430)
Supplement: S1 File — (DOCX) [file pone.0328430.s001.docx]

**S1 File. Supplementary tables (S1_Table–S4_Table)**

**S1 Table.** Results of Cohen Kappa intra reliability test on randomly selected videos for facial action units (AU) (10% of the total number of videos)

| **Facial movement** | **EquiFACs code** | **Cohen Kappa coefficient** |
| --- | --- | --- |
| Ears forward | EAD101 | 0.996 |
| Inner brow raiser | AU101 | 0.993 |
| Blink and Half blink | AU145 + AU47 | 0.999 |
| Upper eyelid lift and white eye increase | AU5 + AD1 | 1.0 |
| Upper lip raiser | AU10 | 0.998 |
| Lip puller | AU113 + AU12 | 1.0 |
| Lower lip depressor | AU16 | 0.999 |
| Chin raiser | AU17 | 0.75 |
| Lip pucker | AU18 | 0.98 |
| Tongue show | AD19 | 1.0 |
| Jaw drop | AU26 | 1.0 |
| Upper Lip Tremor |  | 1.0 |
| Lower Lip Tremor |  | 1.0 |
| Chewing |  | 1.0 |

**S2 Table.** Loadings of facial movements and behavioural variables in the first pre and post-exercise behaviours PCA (PCA1) for 13 French Standardbreds. Data from two time points (pre- and post-exercise) were used to perform the PCA. The table presents the loadings of each variable on the first two principal components (Dimension 1 and Dimension 2).

|  | **Dimension 1** | **Dimension 2** |
| --- | --- | --- |
| ***Facial movements*** | | |
| *Ears* | | |
| Ears forwards: EAD101 | **0.673** | -0.243 |
| *Eyes* | | |
| Inner brow raiser: AU101 | 0.052 | 0.339 |
| Blink and Half blink: AU145 + AU47 | 0.338 | **0.548** |
| Upper eyelid lift and white eye increase: AU5 + AD1 | **0.532** | 0.442 |
| *Mouth and nostrils* | | |
| Upper lip raiser: AU10 | **0.590** | -0.368 |
| Lip puller: AU113 + AU12 | 0.211 | **-0.504** |
| Lower lip depressor: AU16 | **0.637** | -0.021 |
| Chin raiser: AU17 | **0.672** | -0.192 |
| Lip pucker: AU18 | 0.272 | 0.171 |
| Tongue show: AD19 | **0.548** | 0.379 |
| Jaw drop: AU26 | **0.655** | 0.313 |
| Upper Lip Tremor: ULT | **0.656** | -0.461 |
| Chewing | 0.022 | **0.600** |
| Nostril dilatator: AD38 | 0.160 | 0.107 |
| Lower lip depressor: AD160 | 0.432 | -0.374 |
| ***Behaviours*** | | |
| Head turning | -0.086 | **0.631** |
| Headshake | 0.445 | 0.334 |
| Locomotion | 0.238 | 0.395 |
| Paw | -0.253 | 0.331 |
| Hind limb lift | 0.302 | **0.577** |
| Resting leg | 0.383 | -0.100 |
| Weight shifting | 0.068 | 0.217 |

**S3 Table.** Loadings of facial movements and behavioural Variables in the first post-exercise behaviours PCA for 13 French Standardbreds. Data from two time points (pre- and post-exercise) were used to perform the PCA. The table presents the loadings of each variable on the first two principal components (Dimension 1 and Dimension 2).

|  | **Dimension 1** | **Dimension 2** |
| --- | --- | --- |
| ***Facial movements*** | | |
| *Ears* | | |
| Ears forwards: EAD101 | 0.092 | 0.711 |
| *Eyes* | | |
| Inner brow raiser: AU101 | -0.429 | 0.371 |
| Blink and Half blink: AU145 + AU47 | 0.199 | 0.293 |
| Upper eyelid lift and white eye increase: AU5 + AD1 | 0.381 | **0.597** |
| *Mouth and nostrils* | | |
| Upper lip raiser: AU10 | 0.389 | **0.528** |
| Lip puller: AU113 + AU12 | -0.331 | **0.517** |
| Lower lip depressor: AU16 | -0.141 | **0.640** |
| Chin raiser: AU17 | -0.437 | **0.671** |
| Lip pucker: AU18 | 0.440 | -0.139 |
| Tongue show: AD19 | **0.606** | **0.608** |
| Jaw drop: AU26 | 0.474 | **0.564** |
| Upper Lip Tremor: ULT | -0.163 | **0.594** |
| Chewing | -0.222 | 0.476 |
| Nostril dilatator: AD38 | **0.518** | -0.250 |
| Lower lip depressor: AD160 | -0.170 | 0.159 |
| ***Behaviours*** | | |
| Head turning | **-0.586** | 0.332 |
| Headshake | **0.527** | -0.118 |
| Locomotion | **0.613** | -0.074 |
| Paw | **-0.752** | -0.178 |
| Hind limb lift | 0.459 | 0.301 |
| Resting leg | **0.604** | 0.170 |
| Weight shifting | **0.612** | -0.213 |

**S4 Table.** Summary of the model estimates for cortisol, adrenaline, and serotonin levels, with Time Point as a fixed effect. Random effects include Horse and Stable. The table presents estimates (Est/Beta), standard errors (SE), 95% confidence intervals (95% CI), z-values, and p-values for each model. The last column reports the Chi² statistic and p-value comparing each model to the null model (i.e. models with random effects only).

|  | **Est/Beta** | **SE** | | **95% CI** | **z-value** | | | **p** | **Comparison to null model** |
| --- | --- | --- | --- | --- | --- | --- | --- | --- | --- |
| **Cortisol** | | | | | | | | | |
| ***Post*** | -0.348 | 0.0446 | | [-0.435;-0.261] | -7.809 | | | <0.001 | Chi^2^ = 47.1  P < 0.001 |
| ***Post+1*** | -0.213 | 0.0457 | | [-0.303;-0.124] | -4.66 | | | <0.001 |  |
| ***Post+24*** | 0.0153 | 0.0446 | | [-0.0721;0.103] | 0.344 | | | 0.732 |  |
| **Adrenaline** | | | | | | | | | |
| ***Post*** | 1.02 | | 0.331 | [0.375;1.672] | | 3.09 | <0.001 | | Chi^2^ = 33.7  P <0.001 |
| ***Post+1*** | 0.767 | | 0.339 | [0.103;1.432] | | 2.26 | <0.01 | |  |
| ***Post+24*** | -0.677 | | 0.331 | [-1.32; -0.028] | | -2.05 | <0.01 | |  |
| **Serotonin** | | | | | | | | | |
| ***Post*** | 0.532 | | 0.178 | [0.183;0.880] | | 2.99 | <0.001 | | Chi^2^ = 9.94  P <0.05 |
| ***Post+1*** | 0.466 | | 0.178 | [0.117;0.814] | | 2.62 | <0.001 | |  |
| ***Post+24*** | 0.221 | | 0.178 | [-0.128;0.569] | | 1.24 | <0.05 | |  |
