# Supplementary material for: Exploring the association between stress-related hormonal changes, behaviours and facial movements after an interval training exercise in French Standardbred
Source: PLoS One. 2025 Nov 5;20(11):e0328430. doi: 10.1371/journal.pone.0328430 (PMC12588519; doi:10.1371/journal.pone.0328430)
Supplement: Attachment — Submitted filename: Response to Reviewers.docx [file pone.0328430.s003.docx]

We thank the Academic Editor and the reviewers for their careful evaluation of our manuscript and their constructive comments. We have revised the manuscript accordingly. In the following document, each reviewer’s comment is reported in black, and our responses are provided in blue directly below each comment. Line numbers mentioned in our responses refer to the revised manuscript with track changes.

Reviewer #1: Overall this is quite a well conducted study measuring the endocrine response and behaviour post interval training in horses.

However, the problem is the manuscript is written as if there is causality between the physiological effects of exercise and the post exercise behaviour without considering the confounding effect of 1. cross tying horses and 2. restricting movement and rugging hot horses after intense exercise.

The hypotheses could equally be - does cross tying horses cause increase oral related, weight shifting and stress behaviours - or if the horses were also cross tied before exercise (just not stated) - does cross tying hot horses post intense exercise increase oral, shifting, and stress related behaviours.

Although the precise duration and intensity of exercise are not well described, physiologically, horses will have accumulated a large heat burden, continue to sweat and behaviourally would be inclined to want to walk and roll - which is restricted by cross tying them.

The endocrine responses to exercise are not unusual and don't in themselves reflect a negative "stress" perception by the horse, but a normal physiological response to exercise.

The discussion concludes that the behaviour is exercise-induced stress, but this cannot be concluded without the removal of the environmental stressors post exercise e.g. a control group that were cooled down properly and allowed access to a sand roll or unrestricted movement.

The fact that the horses were more aroused post exercise is unsurprising and the degree of arousal reflected in ADR and 5HT being partly related to the increased frequency of behaviours is also expected.

We would like to thank you for your constructive observation. We have clarified in the manuscript that agitation-related behaviours (e.g. pawing, head-turning) did not significantly differ in frequency or duration between pre- and post-exercise. However, at the individual level, horses showing more agitation post-exercise were also those with the largest increases in serotonin and adrenaline. We have carefully nuanced the interpretation of these associations, explicitly acknowledging that causal links between hormones and behaviours cannot be established and that confounding effects of post-exercise management practices (e.g. cross-tying, rugging, restricted movement) may also have contributed. We agree that the endocrine responses observed are not abnormal but likely reflect a normal physiological stress response to exercise, which is necessary for training and not inherently detrimental. In the Abstract and Discussion, we have revised the text to present these findings as a post-exercise arousal response, while highlighting that further studies under alternative recovery conditions would be needed to disentangle exercise-induced effects from management-related influences.

Title/Abstract/Introduction:
Trotting exercise is non specific and presumably refer to interval training - use the most specific term throughout

Thank you for your remark. We have replaced the term “trotting exercise” with “interval training exercise” throughout the manuscript, including in the title, abstract, and methods, to ensure consistency and precision.

Speculation about the bit pressure in the abstract appears unfounded by the dataset

We agree with your point. To avoid over-interpretation, we have removed the sentence from the abstract (former line 40: “This association may reflect a rebound effect of bit pressure on the mouth, which may be greater with greater perceived stress during exercise, and the increased arousal effects of adrenaline, and to a lesser extent, serotonin.”).

Have the authors excluded other causes for agitation post exercise e.g. could be associated with sweating, cooling down etc.

We thank you for this valuable remark. As clarified in the revised version, our investigation of the links between agitation/mouth movements and hormonal changes was performed only on post-exercise behaviours, not on before–after comparisons. Indeed, agitation-related behaviours did not differ significantly in frequency or duration between pre- and post-exercise recordings. Nevertheless, we acknowledge that differences between horses in their level of post-exercise frustration, sweating, or other management-related factors may still have contributed to the individual variability observed in post-exercise behaviours. We have therefore clarified this potential confounding effect in the abstract (lines 40–47) and in the discussion, framing our interpretation as post-exercise arousal responses rather than exclusively exercise-induced stress.

Lines 40-47: “Overall, our findings suggest that a behavioural profile characterised by increased agitation and mouth movements may reflect a post-exercise arousal response in French Standardbreds. These behaviours, in association with hormonal changes, could provide a useful non-invasive tool to assess to assess horses’ response to exercise, and potentially exercise-induced stress. However, further studies are needed to confirm this interpretation, particularly by investigating the potential effects of post-exercise management practices such as cross-tying, which may induce frustration. 

Some grammatical errors e.g. "chronical" instead of chronic, "had a shower", Line 164 "Dosages" observers were validated to use...

We apologise for these mistakes. We have carefully revised the manuscript to correct grammatical errors. Specifically:

- “chronical” has been corrected to “chronic” (line 77).
- “had a shower” has been replaced with “were showered with water” (line 167).
- The sentence on EquiFACS training has been rephrased as: “Observers were validated in the use of the EquiFACS coding system prior to coding the videos.” (line 266)

Methods:

Thank you for these remarks. We have added details in our text accordingly.

Missing details relevant to study
1. duration of turnout in hours (versus frequency per week)

We have added this information in the Methods section (line 131), which now states that horses “were turned out in pasture or paddock for 2 to 4 hours per turnout, at least twice a week.”

2. Training status of the horses i.e. were they race fit or still in training

We have clarified in the Methods that the horses “were race-fit, as they all had either recently raced or were scheduled to race in the near weeks” (line 133).

3. wrt exercise load in the interval session/Duration of intervals (distance or time) - average does not indicate how long the horses spend at maximal or high intensities versus recovery

We have added more detailed information on the exercise sessions in the Methods. The revised text now specifies the number of accelerations, their duration and distance, as well as the duration and distance of the recovery phases between accelerations:

“The exercise was carried out in the morning and was equivalent to interval training, in which horses performed a warm-up at a slow trot, followed by repeated accelerations (2 to 6 per horse, mean ± SD: 3.10 ± 1.19) interspersed with recovery phases at a moderate trot. Each acceleration lasted 4.15 ± 2.36 min (total duration per horse: 9.29 ± 1.83 min) and covered on average 2.54 ± 1.60 km (total acceleration distance: 5.56 ± 0.91 km). The recovery phases between accelerations lasted 3.23 ± 2.16 min on average (total duration: 6.72 ± 3.45 min) and covered 0.76 ± 0.52 km on average (total inter-accelerations distance: 1.73 ± 1.30 km). After the last acceleration, horses cooled down at slow trot before going back to the stable. Overall, horses covered on average 11.5 ± 1.55 km, equivalent to 33.0 ± 5.60 minutes' exercise.” (lines 143-152).

4. Were horses cooled down? Were they scraped as well as rinsed? Were they allowed to roll e.g. in a sand roll following exercise?

We have clarified the post-exercise management procedures in the Methods. The revised text now states: “After exercise, the horses' equipment was immediately removed and they were showered with water, scraped to remove excess water, covered with a blanket and cross-tied in their stalls, as they usually are after exercise. Horses were not allowed to roll after exercise.” (lines 167-169)

5. When were the behaviours and facial movements recorded? Also being cross tied (line 209) is different from being loose in the stall as implied earlier (Line 128 )- clarify

We apologise for this lack of clarity. We have specified in the Methods that “Behavioural and facial movement recordings were recorded while horses were cross-tied, both before and 3 to 5 min post exercise (after showering), to ensure consistency across conditions.” (lines 173–175)

and “The horses were cross-tied in their stalls by means of two ropes or chains, with one side of the halter fastened to a corner of the stall or stall door, as is customary during the preparation and care of the animals.” (lines 255–259)

Discussion -
Line 414 - 415 - in order to conclude as per the Rollkur argument would need to compare cortisol response post interval training in your horses and previous research. Indeed the Rollkur argument agrees with the alternate hypothesis that restriction of hot horses with cross tying port interval training is the stressor - but only if the post exercise cortisol is greater than horses exercise of equivalent intensity and workload.

Thank you for this comment. We agree that our study does not allow us to disentangle the relative contribution of exercise itself versus post-exercise management practices (e.g. cross-tying) to the cortisol response. We have therefore revised the discussion to clarify this limitation. In addition, we would like to point out that our original reference to “constraining postures” referred to conditions during exercise (e.g. Rollkur in dressage) rather than after exercise. To improve clarity and consistency, we have modified this section as follows:

“This increase is most likely to reflect the physiological demands of exercise itself, which represents a normal hormonal response to physical exercise. However, as shown in other studies, stressful contexts or constraining positions can further elevate cortisol responses (1,10,50,51). The elevation in cortisol observed here therefore likely reflects a combination of both physiological and emotional stress.” (lines 497–501)

Lines 431-434 unreferenced and not clearly related to the study.

We have deleted this passage (lines 533) : “However, this hormonal recovery may not fully reflect recovery from physical strain, such as muscle damage, which could be better assessed through additional physiological indicators (e.g., lactate, creatine kinase).”

The hypothesis of the rebound effect from the bit is unfounded from the data in the study. Did the horses in this study have evidence of mouth lesions? Was the degree of rein tension even observed?

We agree that our interpretation regarding a rebound effect from the bit remains a hypothesis. In this study, we did not examine the inside of the horses’ mouths but only the lip commissures, which did not show major lesions. Rein tension was not measured in the present study. To address this point, we have clarified our text to present this interpretation as a plausible hypothesis rather than a conclusion:

"Rein tension, even if not assessed for the present study, is known to be substantial in harness trotting (53), and as reins are directly attached to the bit, the mouth is likely to experience considerable pressure during an interval training exercise. It is therefore plausible that the increased frequency of mouth movements observed post-exercise reflects, at least in part, a release or rebound effect following bit-related pressure." (lines 546–551)

Reviewer #2: Really interesting idea to study, relative perceived effort (RPE) is widely used in sports science as a metric for measuring training load, progress and managing recovery. Correctly applied and interpreted this has beneficial impacts on athlete welfare.
For the horse where it is not possible to use self reflections to gauge the mood-related, emotional, or stress perception of components of the stress response I think this multimodal approach takes into consideration the breadth of the stress response to exercise nicely.
I would love to see a replication of this test to see to what extent individual variation persists, and how these metrics might change through the duration of a training program i.e. is there a period of acclimatisation.

Line 53 - a minor typo - ‘Chronical’ - I think this should say "Chronically"

The limitations and cautions for the results are well-discussed. Overall a really nice study, and one which is really positive for trainers and owners — to know that you can spend time observing your horse (with an educated eye or ethogram guide) to assess their response to training is a really important message at this time when welfare in horse sport is so crucial.

Thank you for your encouraging comments. We are glad that you found the multimodal approach relevant and agree that replicating this study across different stages of training would be valuable to explore potential acclimatisation effects and the persistence of individual variation. We have corrected the typo you mentioned (“chronical” changed to “chronic,” line 77). We also appreciate your positive feedback regarding the welfare implications of our findings, and we have made sure to emphasise this point more clearly in the Discussion and Conclusion.
